# Copy Number Variation in Acetolactate Synthase Genes of Thifensulfuron-Methyl Resistant *Alopecurus aequalis* (Shortawn Foxtail) Accessions in Japan

**DOI:** 10.3389/fpls.2017.00254

**Published:** 2017-03-02

**Authors:** Satoshi Iwakami, Yoshiko Shimono, Yohei Manabe, Masaki Endo, Hiroyuki Shibaike, Akira Uchino, Tohru Tominaga

**Affiliations:** ^1^Graduate School of Agriculture, Kyoto UniversityKyoto, Japan; ^2^Crop Production Systems Division, NARO Agricultural Research CenterTsukuba, Japan; ^3^Faculty of Life and Environmental Sciences, University of TsukubaTsukuba, Japan; ^4^Plant Genome Engineering Research Unit, Institute of Agrobiological Sciences, National Agriculture and Food Research OrganizationTsukuba, Japan; ^5^National Institute for Agro-Environmental SciencesTsukuba, Japan; ^6^Central Region Agricultural Research Center, National Agriculture and Food Research OrganizationTsu, Japan

**Keywords:** shortawn foxtail, ALS inhibitor, herbicide resistance, target-site resistance, copy number variation

## Abstract

Severe infestations of *Alopecurus aequalis* (shortawn foxtail), a noxious weed in wheat and barley cropping systems in Japan, can occur even after application of thifensulfuron-methyl, a sulfonylurea (SU) herbicide. In the present study, nine accessions of *A. aequalis* growing in a single wheat field were tested for sensitivity to thifensulfuron-methyl. Seven of the nine accessions survived application of standard field rates of thifensulfuron-methyl, indicating that severe infestations likely result from herbicide resistance. Acetolactate synthase (ALS) is the target enzyme of SU herbicides. Full-length genes encoding ALS were therefore isolated to determine the mechanism of SU resistance. As a result, differences in *ALS* gene copy numbers among accessions were revealed. Two copies, *ALS1* and *ALS2*, were conserved in all accessions, while some carried two additional copies, *ALS3* and *ALS4*. A single-base deletion in *ALS3* and *ALS4* further indicated that they represent pseudogenes. No differences in ploidy level were observed between accessions with two or four copies of the *ALS* gene, suggesting that copy number varies. Resistant plants were found to carry a mutation in either the *ALS1* or *ALS2* gene, with all mutations causing an amino acid substitution at the Pro197 residue, which is known to confer SU resistance. Transcription of each *ALS* gene copy was confirmed by reverse transcription PCR, supporting involvement of these mutations in SU resistance. The information on the copy number and full-length sequences of *ALS* genes in *A. aequalis* will aid future analysis of the mechanism of resistance.

## Introduction

Herbicides that inhibit acetolactate synthase (ALS) cause depletion of branched chain amino acids such as valine, leucine and isoleucine, leading to plant death (Duggleby et al., 2008; Yu and Powles, 2014). However, recurrent use of these herbicides has resulted in the rapid evolution of herbicide resistance in weeds. Globally, resistance to ALS inhibitors has been reported in 159 weed species (Heap, 2016). ALS inhibitors are categorized into five groups based on their chemical structure: sulfonylurea (SU), triazolopyrimidine (TP), pyrimidinylthiobenzoate (PTB), imidazolinone (IMI), and sulfonylaminocarbonyltriazolinone (SCT). In Japan, SUs are predominantly used due to their excellent crop safety, broad spectrum of weed control and low toxicity to animals. Thus, most cases of resistance to ALS-inhibiting herbicides reported in Japan, such as *Monochoria vaginalis* (Ohsako and Tominaga, 2007), *Schoenoplectus juncoides* (Uchino et al., 2007), and *Sagittaria trifolia* (Iwakami et al., 2014b), have evolved under SU selection (Uchino et al., 2016).

Resistance of weeds to herbicides is caused by target-site and /or non-target-site resistance mechanisms (Powles and Yu, 2010). Target-site resistance involves alterations to the target site such as overproduction and amino acid substitution of the target protein, while non-target-site resistance includes all other mechanisms such as enhanced herbicide metabolism, restricted herbicide translocation and reduced herbicide uptake. In the case of ALS inhibitor resistance, target-site resistance resulting from amino acid substitution frequently occurs (Yu and Powles, 2014). A substitution at one of eight amino acid residues in the ALS protein sequence of *Arabidopsis thaliana*, Ala122, Pro197, Ala205, Asp376, Arg377, Trp574, Ser653, or Gly654, was found to cause resistance to ALS inhibitors (Yu and Powles, 2014).

Accumulating evidence further suggests that copy numbers of genes encoding herbicide targets also has an effect on the evolution of herbicide resistance. For example, in a polyploid, a single mutation in one homoeologous copy encoding a target enzyme tends to confer lower levels of herbicide resistance compared to diploid plants carrying the same mutation in a single-copy gene (Yu et al., 2013). The locus of a mutated copy among multiple homoeologous copies of genes encoding herbicide targets could also influence the degree of herbicide resistance (Ostlie et al., 2015). In addition, within-species copy number variation (CNV) was previously observed in the gene encoding the glyphosate target enzyme, 5-enolpyruvoylshikimate-3-phosphate synthase (EPSPS), of which a higher copy number was associated with glyphosate resistance (Sammons and Gaines, 2014). It is therefore important to determine copy numbers of genes encoding target enzymes and identify the particular copy carrying a resistance-conferring mutation.

*Alopecurus aequalis* Sobol. (shortawn foxtail), family Poaceae, is a diploid species distributed throughout Europe, temperate Asia and North America (Cope, 1982). Its strong tillering capacity allows it to out-compete wheat seedlings, causing yield losses of more than 50 % (Guo et al., 2015a). In Japan, *A. aequalis* is a major weed in barley and wheat fields. Since the early 1990s, management has relied on postemergence application of thifensulfuron-methyl, a SU herbicide. However, resistance to thifensulfuron-methyl was confirmed in 2004, after seven consecutive years of herbicide treatment (Uchikawa et al., 2005). Resistance of *A. aequalis* to ALS inhibitors was also recently reported in China (Guo et al., 2015b, 2016; Xia et al., 2015). Although mutations in *ALS* gene causing Pro197Arg, Pro197Thr, or Trp574Leu have been reported, it remains unknown whether these populations are homozygous or heterozygous at the *ALS* loci and which copies of the *ALS* gene carry a mutation. In this study, we therefore determined the full-length sequences and copy numbers of *ALS* genes in *A. aequalis*, and identified mutations in resistant plants thought to be responsible for resistance to thifensulfuron-methyl. CNV in *ALS* genes in *A. aequalis* was also examined.

## Materials and Methods

### Plant Materials

Seeds of nine *A. aequalis* plants (hereafter referred to as accessions) were collected in May 2012 from a ∼1-ha wheat field in Kumamoto City, Kumamoto Prefecture, Japan (**Table 1**). The field was under a rice–wheat cropping system and was severely infested with *A. aequalis* after thifensulfuron-methyl treatment. Single plants of each accession were grown in a greenhouse and self-pollinated once and the seeds assayed for thifensulfuron-methyl sensitivity. Nucleic acids were also extracted from the seedlings for gene cloning and Southern blot analysis. Individual seedlings from each accession were self-pollinated and used for gene expression and genotyping analyses. An *A. japonicus* accession was also used in this study, and was collected in May 2012 from a wheat field in Mifune, Kumamoto Prefecture, Japan.

**Table 1:** *ALS* gene copy numbers and mutations in *Alopecurus aequalis* accessions.

Accession	Resistance status^∗^	Copy number status		Substitution at Pro197	
		Copy number	Identified gene	Gene	Substituted amino acid
Sugi-1	S	2	*ALS1, ALS2*	–	–
Sugi-2	R	2	*ALS1, ALS2*	*ALS1*	Ser
Sugi-3	R	2	*ALS1, ALS2*	*ALS2*	Leu
Sugi-5	R	4	*ALS1, ALS2, ALS3, ALS4*	*ALS1*	Leu
Sugi-11	R	4	*ALS1, ALS2, ALS3, ALS4*	*ALS1*	Leu
Sugi-14	R	4	*ALS1, ALS2, ALS3, ALS4*	*ALS1*	Leu
Sugi-18	R	2	*ALS1, ALS2*	*ALS1*	Thr
Sugi-24	S	2	*ALS1, ALS2*	–	–
Sugi-29	R	4	*ALS1, ALS2, ALS3, ALS4*	*ALS1*	Thr

*^∗^R, resistant; S, sensitive.*

### Thifensulfuron-Methyl Dose-Response Assay

Seeds were germinated on 0.6% agar plates in a growth chamber at 25/15°C (day / night) with a 12 h photoperiod. After germination, six seedlings per accession were transplanted in a cell tray filled with soil and kept at an ambient temperature in a vinyl greenhouse at Kyoto University during winter 2016 (January to March). At the 3–4 leaf stage, plants were treated with a commercial formulation of thifensulfuron-methyl (Harmony, DuPont, Tokyo, Japan) at 0, 1/3, 1 and 3× the recommended rate (75 g a.i. ha^-1^), respectively. Three weeks after thifensulfuron-methyl application, the dry weights of shoots were measured to compare relative growth among the accessions. The experiment was repeated twice with three replications. Results of a single experiment are shown since they were similar between experiments.

Statistical analyses were performed using square root transformed data. One-way ANOVA with Dunnett’s post-test was performed using R version 3.3.1 (R core team, 2016) to determine differences in sensitivity of the Sugi-1 accession with all other accessions.

### Isolation and Sequencing of *ALS* Genes

Isolation and sequencing analysis of *ALS* genes was carried out using plants of all nine accessions grown in a greenhouse. Green leaves were harvested at the heading stage, snap-frozen and stored at –80°C until use. RNA was isolated using the RNeasy Plant Mini Kit (Qiagen, CA, USA) and genomic DNA removed using the TURBO DNA-*free* Kit (Life Technologies, CA, USA). Complementary DNA (cDNA) was synthesized from the RNA using the SMART RACE cDNA Amplification Kit (TaKaRa, Otsu, Japan), and extracted using the DNeasy Plant Mini Kit (Qiagen).

Partial *ALS* genes of all accessions were amplified from genomic DNA samples using KOD FX (Toyobo, Osaka, Japan) with primers designed based on *ALS* genes from other grass species: forward primer, 5′-AAGGGCGCSGACATCCT-3′; reverse primer, 5′-ATCTGCTGYTGGATGTCCTT-3′. Amplicons were subjected to direct sequencing using BigDye Terminator V3.1 on a 3130xl Genetic Analyzer (Applied Biosystems, CA, USA). Fragments of *ALS* genes amplified from accessions Sugi-5 and Sugi-24 were cloned into pGEM-T Easy (Promega, Madison, WI, USA) and the inserts sequenced.

The 5′ and 3′ regions of each *ALS* gene fragment from Sugi-5 were determined by Rapid Amplification of cDNA Ends (RACE) according to either the manufacturer’s instructions for the SMART RACE cDNA Amplification Kit, Thermal Asymmetric Interlaced (TAIL) PCR (Liu and Whittier, 1995) or fusion primer and nested integrated (FPNI) PCR (Wang et al., 2011). The gene-specific primers used for PCR are listed in **Table 2**. The resulting amplicons were then subjected to direct sequencing or cloned and sequenced as described above. Full-length *ALS* gene sequences were amplified using KOD FX with the primers listed in **Table 3** and the sequences confirmed by direct sequencing.

**Table 2:** Primers used for 5′- and 3′-RACE cloning of *ALS* genes from *A. aequalis*.

	Primer sequence		
Gene	1st PCR	2nd PCR	3rd PCR
**5′-RACE**			
*ALS1*	5′-AGGCTTCCTGAATGACGCGGGGAATG-3′	5′-GGGTGACCTCGACTATGGGCGTCTCT-3′	–
*ALS2*	5′-AGGCTTCCTGAATGACGCGGGGGATA-3′	5′-GTGACCTCGACTATGGGCGTCTCC-3′	–
**3′-RACE**			
*ALS1*	5′-CAATGGAGATCCACCAGGCGCTCACA-3′	5′-CGTCTCCGCGCTCGCCGATGCTCTT-3′	–
*ALS2*	5′-CAATGGAGATCCACCAGGCGCTCACG-3′	5′-GATCGGCACGGACGCCTTCCAG-3′	–
**FPIN PCR (5′ Isolation)**			
*ALS3, ALS4*	5′-GTGATGGAGCGGGTGACCTCCA-3′	5′-TCCACTATGGGCGTCTCCTGGAAAG-3′	5′-GCGTATCCGGACGCCGCGAAG-3′
**TAIL PCR (5′ Isolation)**			
*ALS1*	5′-AGGCTTCCTGAATGACGCGGGGAATG-3′	5′-GGGTGACCTCGACTATGGGCGTCTCT-3′	5′-ACCATGGGGATGGAGTCGAGA-3′
**TAIL PCR (3′ Isolation)**			
*ALS3, ALS4*	5′-CTCACGCCGCGCTCTCAACTCT-3′	5′-GATCGGCACGGACGCTTTCCAG-3′	5′-GGAGGACATTCCCCGCGTCATC-3′

**Table 3:** Primers used for amplification of full-length *ALS* genes from *A. aequalis*.

Gene	Forward	Reverse
*ALS1*	5′-CAATAAAAATCTCATGCCCGT-3′	5′-CATGGTTCACAGTTGACCACA-3′
*ALS2*	5′-ACGCTCGCATAAAAAGCCA-3′	5′-GTCCTCTAGGTCGAGCTCTTGATT-3′
*ALS3* and *ALS4*	5′-CACACACTCAGATAAAAAGCCA-3′	5′-AGGTCGAGCTCTTGCTGAAG-3′

### Phylogenetic Analysis of ALS Protein Sequences

Amino acid sequences of plant *ALS* genes were obtained from GenBank, the MSU Rice Genome Annotation Project^1^ or Phytozome v11.0^2^, and a phylogenetic tree generated using MEGA6 (Tamura et al., 2013). Sequences were aligned using ClustalW, and the JTT matrix-based method used to compute evolutionary distances.

### Analysis of Genetic Inheritance of the *ALS3* and *ALS 4* Genes

Seedlings of accession Sugi-5 were self-pollinated twice using the single-seed descent method in the greenhouse at Kyoto University. DNA was then extracted from shoot tissues using the One Step Method according to instructions provided by the manufacturer of KOD FX polymerase. PCR was conducted using KOD FX with primers designed from conserved regions of the *ALS3* and *ALS4* genes (**Table 3**). The amplicons were then subjected to direct sequencing to determine which sequence is carried in the progeny.

### Southern Blot Analysis

Genomic DNA was extracted from shoots of individual plants of single-self-pollinated Sugi-5 and Sugi-24 seedlings using a Plant Genomic DNA Kit (Tiangen, Beijing, China) according to the manufacturer’s instructions. Ten microgram of DNA was digested with *Eco*RV and *Hind*III (New England BioLabs Japan, Tokyo, Japan), then separated electrophoretically on 1% agarose gels and transferred to positively charged nylon membranes (Roche, Basel, Switzerland). A probe for the *ALS* gene was prepared by PCR using the PCR DIG Probe Synthesis Kit (Roche) with primers (forward, 5′-CTTTTGCAAGCAGGTCCAAG-3′; reverse, 5′-ATCCCCATCAATGTCAACAAC-3′) and the *ALS1* PCR product as a template. Hybridization was conducted according to the DIG Application Manual (Roche) and the signals detected using CDP-Star (Roche) on ChemiDoc Touch (Bio-Rad, Irvine, CA, USA).

### Flow Cytometric Analysis

Ploidy levels were analyzed using an Attune Flow Cytometer (Applied Biosystems). *A. aequalis* leaves were chopped using a razor blade in extraction buffer (Galbraith et al., 1983). After filtration using a CellTrics^®^ 20-μm filter (Sysmex, Kobe, Japan), propidium iodide (25 mg/l) was added to stain the DNA. The nuclei suspension was then analyzed by laser excitation at 488 nm.

### Transcription of *ALS* Genes

Plants were grown in a growth chamber at 25°C with a 12 h photoperiod for 20 days after germination. Shoot and root RNA was extracted as described above from three Sugi-5 and Sugi-24 plants, respectively. RNA samples (1 μg) were reverse-transcribed using ReverTra Ace (Toyobo) and PCR amplification conducted using rTaq DNA polymerase (Toyobo) with primers listed in **Table 4**. PCR conditions were as follows: 94 °C for 2 min followed by 35 cycles of 94°C for 30 s, 64°C for 30 s, 72°C for 30 s, and ending with 72°C for 5 min. The PCR products were run on 1% agarose gel and stained with ethidium bromide. Experiments were conducted twice using RNA samples from distinct individuals. Similar results were obtained in each experiment.

**Table 4:** Primers used for analysis of *ALS* gene expression.

Gene	Forward	Reverse	Expected band size (bp)
*ALS1*	5′-GCCAACCCAGGGGTCACA-3′	5′-CATGGTTCACAGTTGACCACA-3′	514
*ALS2*	5′-CGTTGCAGGGCTTGAACA-3′	5′-GTCCTCTAGGTCGAGCTCTTGATT-3′	786
*ALS3* and *ALS4*	5′-GCCAACCCAGGTGTAACC-3′	5′-AGGTCGAGCTCTTGCTGAAG-3′	464

## Results

### Thifensulfuron-Methyl Sensitivity

**FIGURE 1:**
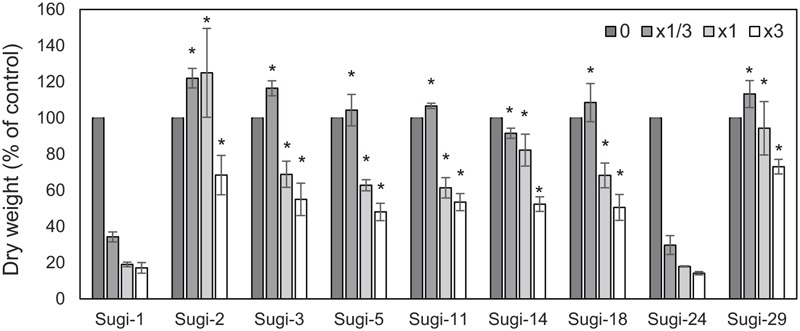
**Thifensulfuron-methyl responses of the *Alopecurus aequalis* accessions.** Each accession was treated with 0, 1/3, 1, or 3× the recommended field application rate of thifensulfuron-methyl. Shoot dry weights are shown as percentages of untreated controls. Bars indicate the standard error (*n* = 3). Dry weights of accession Sugi-1 were compared with remaining accessions using Dunnett’s test. ^∗^*p* < 0.01.

**Figure 1** shows the results of the thifensulfuron-methyl sensitivity assay in nine *A. aequalis* accessions collected from a severely infested wheat field. Growth of two accessions, Sugi-1 and Sugi-24, was severely affected by application of 1/3× the recommended field rate, with dry weights reaching only 35% that of the untreated control. Moreover, plants of both accessions died under treatment with 1 and 3× the recommended field rate. These accessions were therefore considered thifensulfuron-methyl susceptible. In comparison, the remaining seven accessions survived application of 1× the recommended field rate, achieving dry weights of >60% that of the untreated control. Even under 3× the field rate, these accessions exhibited dry weights ≥50% that of the untreated control, indicating thifensulfuron-methyl resistance. All plants from each accession responded to each dose of thifensulfuron-methyl in a similar manner, with no segregation of resistance.

### Isolation and Analysis of *ALS* Genes

**FIGURE 2:**
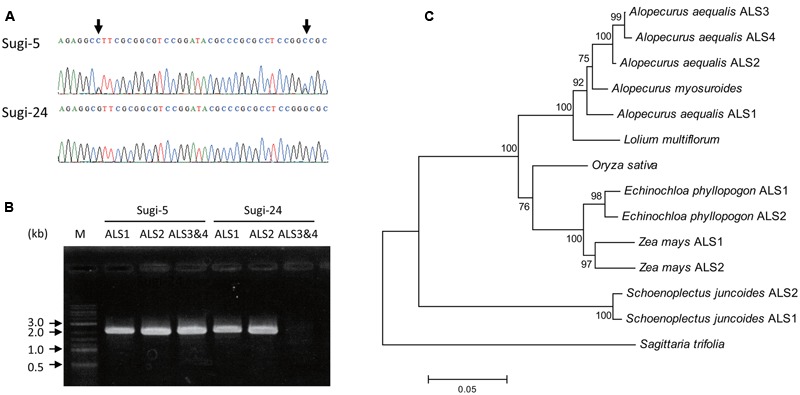
***ALS* genes in *A. aequalis*. (A)** Chromatograms of DNA sequences of an *ALS* gene fragment. Arrows indicate double peaks. **(B)** Amplification of full-length *ALS* genes from accessions Sugi-5 and Sugi-24. M, DNA size standard. **(C)** Phylogenetic tree of the ALS. Numbers at each node indicate the reliability of branches estimated by bootstrap analysis with 1,000 replicates. Accession numbers for each sequence are as follows: *Alopecurus myosuroides*, AJ437300; *Lolium multiflorum*, AAG30931.1; *Oryza sativa*, LOC_Os02g30630.2; *Echinochloa phyllopogon* ALS1, AB636580.1; *Echinochloa phyllopogon* ALS2, AB636581.1; *Zea mays* ALS1, GRMZM2G143008_T01; *Zea mays* ALS2, GRMZM2G143357_T01; *Schoenoplectus juncoides* ALS1, BAE97675.1; *Schoenoplectus juncoides* ALS2, BAE97677.1; *Sagittaria trifolia*, AB301496.1. In the case of rice ALS, an additional ALS-like sequence, LOC_Os04g32010.1, was found in the MUS database; however, this sequence was excluded due to its low level of expression and the large number of unconserved residues among grass ALSs.

Partial fragments of *ALS* genes were amplified from genomic DNA of each accession and subjected to direct sequencing. More than one peak was observed at some nucleotide positions in chromatograms of each accession, suggesting the existence of multiple copies of the *ALS* gene. Interestingly, the chromatogram patterns differed among accessions, with double peaks observed in some accessions, and very sharp single peaks in others (**Figure 2A**), suggesting CNV of the *ALS* genes.

**FIGURE 3:**
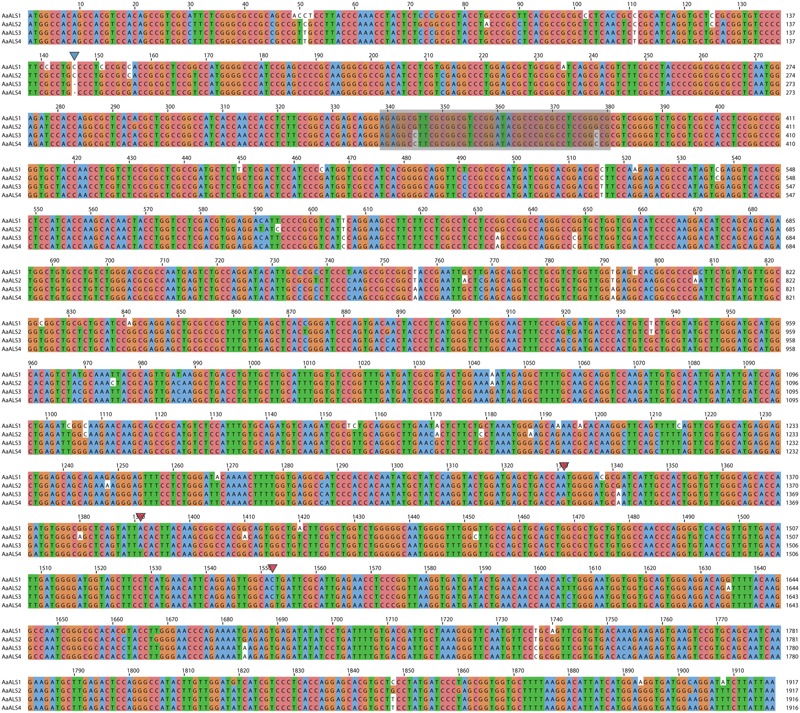
**Sequence alignment of coding sequences of *ALS* genes in *A. aequalis*.** The gray box (+339 to +380) represents the region shown in the chromatogram in **Figure 2A**. Red arrows indicate the positions of SNPs between *ALS3* and *ALS4*, and the blue arrow indicates the deletion that caused a frameshift in *ALS3* and *ALS4*.

PCR amplification of the 3′ and 5′ regions of the partial sequences resulted in the isolation of two copies of the *ALS* gene (*ALS1* and *ALS2*) from accession Sugi-24 and four copies (*ALS1, ALS2, ALS3*, and *ALS4*) from accession Sugi-5 (accession numbers: LC200800 to LC200803) (**Figures 2, 3**). The double peaks observed in direct sequencing of the Sugi-5 PCR product (**Figure 2A**) therefore represented *ALS3* and *ALS4*. The *ALS2* gene sequence was identical to that of the *A. aequalis ALS* gene sequence previously deposited in GenBank (JQ743908.1). The *ALS3* and *ALS4* genes each contained a single base deletion in a region containing four consecutive Cs at positions +146 to +149 (**Figure 3**), respectively, which would result in aberrant protein sequences. Thus, *ALS3* and *ALS4* are thought to be pseudogenes that share 99.5% identity with only three single nucleotide polymorphisms (SNPs) in the coding sequence (**Figures 2C**, **3**). Polymorphisms between *ALS3* and *ALS4* were limited even in untranslated regions (UTRs) and the promoter region; no polymorphisms were observed in the 3′-UTR (157 bp), while four SNPs and three bp deletions were revealed in the promoter and 5′-UTR, respectively (603 and 600 bp for *ALS3* and *ALS4*, respectively) (Supplementary Figure S1). This discovery of a limited number of polymorphisms between *ALS3* and *ALS4* precluded the design of gene-specific primers. None of the *A. aequalis ALS* genes contained introns, as previously observed in *ALS* genes in Poaceae (Iwakami et al., 2012). The predicted protein sequences of ALS1 and ALS4 exhibited high homology with those of ALS proteins from *A. myosuroides* (AJ437300) at 96.2 to 97.5% identity (**Figure 2C**). Furthermore, ALS3, which shared 99.5 % identity with ALS4, exhibited 95.8 and 98.9% identity with ALS1 and ALS2, respectively.

The possibility that *ALS3* and *ALS4* represent alleles of a single gene, rather than two different genes, was subsequently investigated since the two were found to be highly homologous. To do so, sequences were investigated in the self-pollinated progeny of next-generation plants carrying both sequences. Full-length sequences of *ALS3* and *ALS4* were amplified and the DNA fragments directly sequenced. A chromatogram of all the progeny (more than 30 individuals) showed a double peak at the three polymorphic sites between the *ALS3* and *ALS4* sequences only. This finding confirms that all the progeny carried both the *ALS3* and *ALS4* sequences, contradicting the suggestion that they represent alleles and providing strong evidence that they represent two different genes.

The results of Southern blot analysis supported the differences in copy number among accessions. In Sugi-24, which carried two copies (*ALS1* and *ALS2*), two signals were detected both in the *Eco*RV and *Hind*III digests (Supplementary Figure S2). In contrast, three signals were detected in the *Hind*III digest from Sugi-5, in which an additional two copies were found, although no differences in hybridization patterns of the *Eco*RV digests were observed. In both accessions, lower molecular weight signals in the *Eco*RV digest were thought to represent *ALS2*, considering the restriction sites of *Eco*RV (Supplementary Figure S2C).

The full-length sequences of *ALS* genes amplified from all nine accessions were subsequently sequenced. *ALS1* and *ALS2* were observed in all accessions analyzed so far (48 accessions from eight fields, data not shown), while some were found to carry additional *ALS3* and *ALS4* copies (**Table 1**). All sequences of each gene were identical, except at the codon corresponding to Pro197, mutations of which are known to confer resistance to several SU herbicides. Here, all of the thifensulfuron-methyl resistant accessions carried mutations at Pro197, with substitutions of Ser, Leu or Thr identified in *ALS1* (**Table 1**). However, in Sugi-3, a Leu substitution was observed in the Pro197 of *ALS2*.

### Ploidy Levels

**FIGURE 4:**
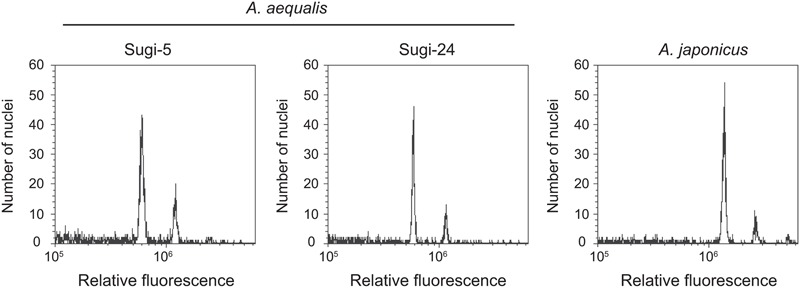
**Relative fluorescence intensities from flow cytometry analysis**.

Two nuclei peaks were detected in flow cytometric analyses of two accessions of *A. aequalis* and one accession of *A. japonicus* (**Figure 4**). The fluorescence intensities of the first and second peaks were the same in the *A. aequalis* accessions, with two and four copies of the *ALS* genes. In contrast, peak intensities of the closely related tetraploid *A. japonicus* (Xu et al., 2014) were double those of the *A. aequalis* accessions, suggesting that DNA contents of single nuclei from two- and four-copy *A. aequalis* accessions are equal and approximately half that of *A. japonicus*.

### Transcription of the *ALS* Genes

To confirm transcription of the *ALS* genes in seedlings from accessions carrying two or four copies, reverse transcription (RT)-PCR was conducted. PCR amplification could not distinguish *ALS3* and *ALS4* due to the lack of nucleotide polymorphisms. Thus, primers targeting the conserved region between the two genes were designed in order to amplify the area containing the polymorphisms. The resulting PCR product was subjected to direct sequencing to confirm which genes were transcribed.

**FIGURE 5:**
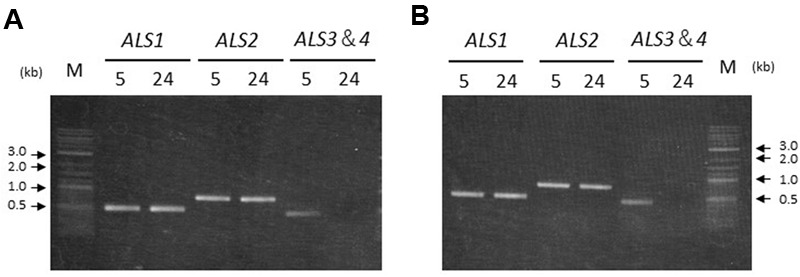
**Transcription of *ALS* genes in two *A. aequalis* accessions.** Labels 5 and 24 represent the Sugi-5 and Sugi-24 accessions, respectively. The primers do not discriminate between the *ALS3* and *ALS4* genes. M, DNA size standard. **(A)** Seedling shoots. **(B)** Seedling roots.

*ALS1* and *ALS2* were transcribed both in the shoots and roots in accessions carrying two or four copies of the *ALS* genes; namely, Sugi-24 and Sugi-5, respectively (**Figure 5**). *ALS3* and *ALS4* DNA fragments were subsequently amplified from Sugi-5 shoot and root template cDNA. Sequencing of the PCR products revealed transcription of both the *ALS3* and *ALS4* genes. No DNA fragments of any of the *ALS* genes were detected in controls without reverse transcriptase (data not shown).

## Discussion

In the present study, thifensulfuron-methyl resistant *A. aequalis* accessions were found to carry a mutation at the Pro197 residue in either the *ALS1* or *ALS2* gene. All amino acid substitutions identified in this study are known to confer SU resistance (Yu and Powles, 2014). Moreover, both the *ALS1* and *ALS2* genes were found to be transcribed in seedlings of *A. aequalis*, supporting involvement of this mutation in thifensulfuron-methyl resistance. However, it remains to be determined whether the mutation site or number of mutated loci influences the degree of resistance. More detailed characterization of these accessions will therefore be carried out in the future.

The number of *ALS* gene sequences differed among the nine accessions. Differences in the copy number of genes can result from CNV or from different ploidy levels within a species. In the present study, analysis of ploidy level revealed no differences in the amount of nuclear DNA among accessions. It is therefore unlikely that the higher-copy number accessions are tetraploid, although ploidy level variation within a single species has been reported in *Alopecurus* spp. including *A. aequalis* (Sieber and Murray, 1980; Koul and Gohil, 1990). The two highly similar DNA sequences of the higher–copy number accessions (*ALS3* and *ALS4* sequences) suggested that they might represent alleles of a single gene rather than two different genes. However, analysis of self-pollinated progeny revealed that all offspring carried both sequences, confirming that they are not alleles. Meanwhile, Southern blot analysis indicated two or three bands rather than four, which is consistent with different restriction enzymes (data not shown). This discrepancy can be explained by the hypothesis that *ALS3* and *ALS4* are tandemly duplicated and tightly linked on the same chromosome, supporting the observation that no three-copy individuals were present among 48 accessions in eight fields (data not shown). The occurrence of CNV in *ALS* genes has also been suggested in the tetraploid *A. japonicus* (Feng et al., 2016), with three copies identified in one population and an additional copy in another. This additional copy might represent an ortholog of *ALS3* and *ALS4* identified in this study, since one of the subgenomes of the hexaploid *A. japonicus* is thought to have been derived from *A. aequalis* (Matumura, 1968).

The CNV revealed in this study is unlikely to influence the evolution or level of resistance since both additional copies were thought to be pseudogenes without functions. However, copy number amplification of functional *ALS* genes could have resulted in ALS inhibitor resistance as previously observed in cultured cells (Odell et al., 1990), although resistance levels would be lower than that of target-site resistance resulting from amino acid substitution. Activity of plant ALS is subject to feedback inhibition from branched chain amino acids (Duggleby and Pang, 2000) and is not necessarily positively correlated with protein levels. In line with this, overproduction of ALS was found to result in only a slight decrease in ALS inhibitor sensitivity (Odell et al., 1990; Whaley et al., 2007).

The discovery of CNV in the *ALS* gene warrants further analysis of copy numbers in genes encoding herbicide targets, especially *EPSPS*. *ALS* genes are frequently used as an internal control in real-time PCR analysis of copy numbers of *EPSPS* genes, since *ALS* copy number was previously thought to be stable among individuals. However, this study revealed that the *ALS* copy number is, in fact, unstable. Selection of genes for use as an internal control should therefore be made carefully, and copy number amplification of genes of interest validated by other methods such as Southern blotting or fluorescence *in situ* hybridization as in Gaines et al. (2010), Jugulam et al. (2014), and Dillon et al. (2016). Alternatively, additional real-time PCR using a second internal control gene such as the cinnamoyl-CoA reductase gene (Salas et al., 2012, 2015) should also be performed for validation.

One interesting finding of this study was the presence of various mutation patterns in accessions randomly collected from a small field. Since *A. aequalis* is a self-fertile species, one might expect resistant plants in a single field to be derived from a single progenitor, as previously seen in resistant populations of *M. vaginalis* in Japan (Imaizumi et al., 2008). However, the discovery of four resistant genotypes suggests that the evolution of resistance occurred multiple independent times. Further research is now needed to determine how these resistance alleles evolved.

Despite the findings, the present results do not exclude the possible involvement of a non-target-site resistance mechanism. Non-target-site resistance is a major mechanism in *A. myosuroides*, which is a close outcrossing relative of *A. aequalis* (Délye et al., 2011). Non-target-site resistance to ALS inhibitors is often associated with enhanced activity of cytochrome P450 (Yuan et al., 2007). Recently, we discovered that overexpression of herbicide-metabolizing P450 genes is associated with resistance to ALS inhibitors in *Echinochloa phyllopogon*, a self-pollinating Poaceae species (Iwakami et al., 2014a). It is therefore possible that resistance evolved via a similar mechanism in *A. aequalis*.

## Author Contributions

SI, YS, YM, AU, and TT designed the experiments; SI, YS, YM, ME, and HS performed the experiments and analyzed the results; and SI, YS, AU, and TT wrote and approved the manuscript.

## Conflict of Interest Statement

The authors declare that the research was conducted in the absence of any commercial or financial relationships that could be construed as a potential conflict of interest.

The reviewer PTFM and handling Editor declared their shared affiliation, and the handling Editor states that the process nevertheless met the standards of a fair and objective review.

## Footnotes

1 http://rice.plantbiology.msu.edu/index.shtml

2 https://phytozome.jgi.doe.gov/pz/portal.html
