# Supplementary material for: Copy Number Variation in Acetolactate Synthase Genes of Thifensulfuron-Methyl Resistant Alopecurus aequalis (Shortawn Foxtail) Accessions in Japan
Source: Front Plant Sci. 2017 Mar 2;8:254. doi: 10.3389/fpls.2017.00254 (PMC5332366; doi:10.3389/fpls.2017.00254)
Supplement: Supplementary file 1 [file Presentation_1.PDF]

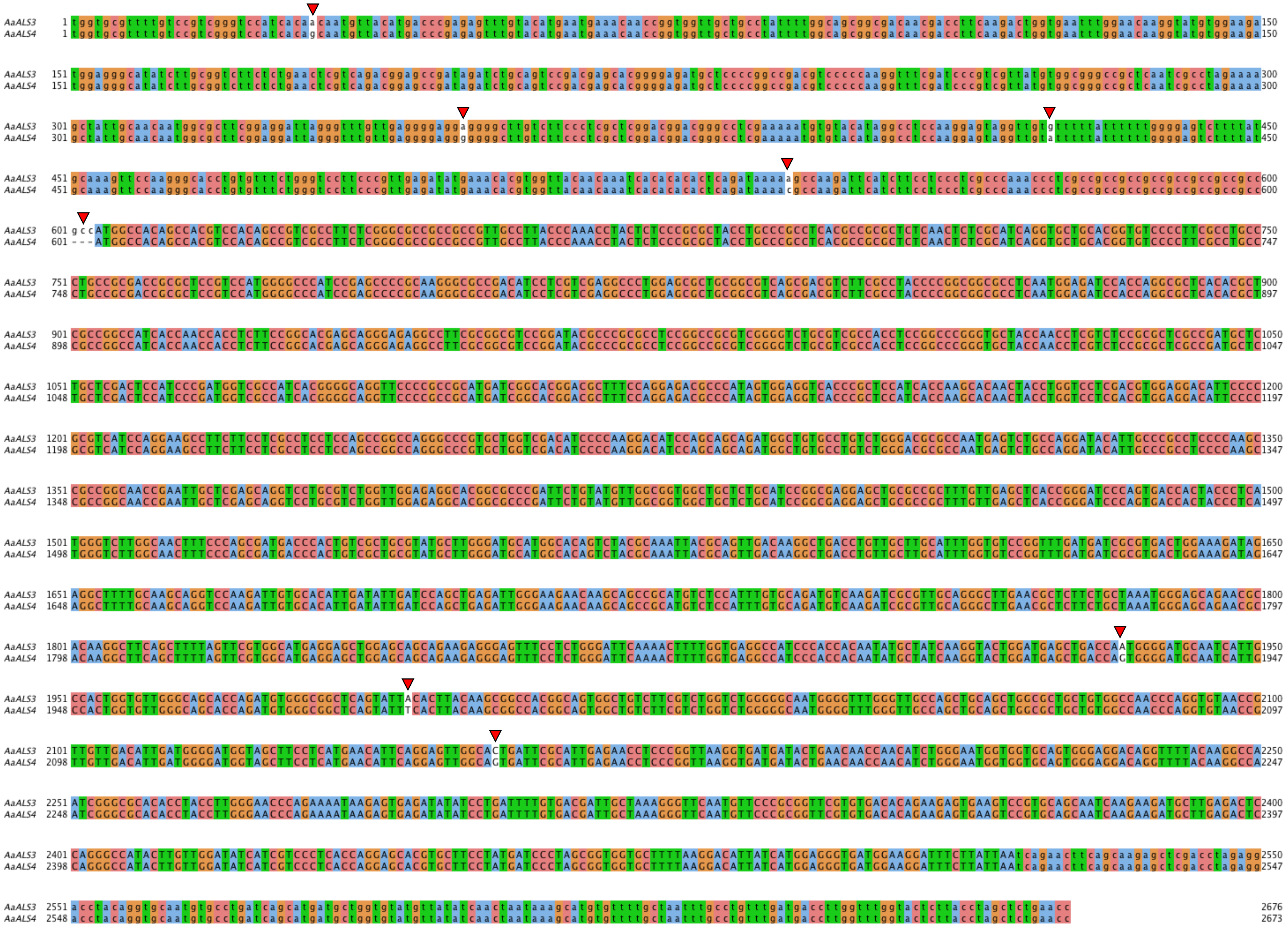

Supplementary Figure 1. Sequence alignment of *ALS3* and *ALS4* genes in *A. aequalis*

Upper and lowercase letters indicate coding and flanking sequences, respectively. Arrows indicate where polymorphisms were observed.

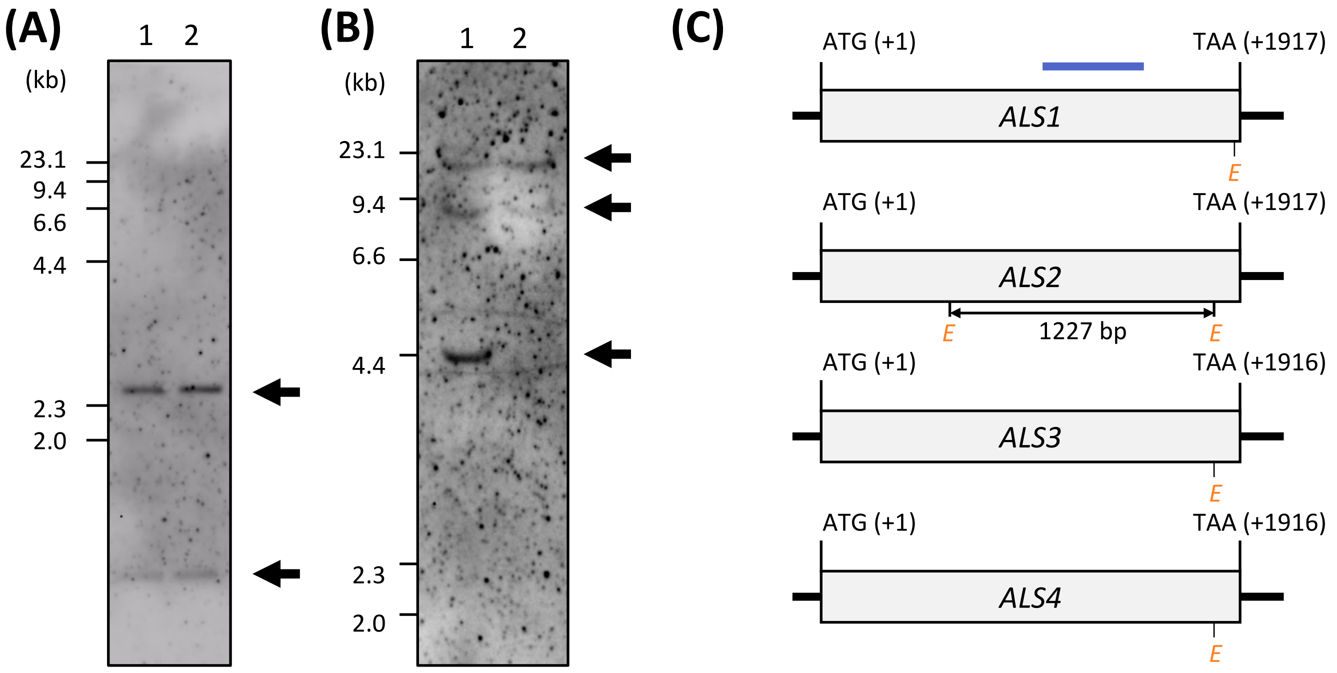

Supplementary Figure 2. Comparison of *ALS* gene copy numbers in two *A. aequalis* accessions by Southern blot analysis

Lane 1, Sugi-5; Lane 2, Sugi-24. (**A**) DNA digested with *Eco*RV. (**B**) DNA digested with *Hind*III. Arrows indicate positions of detected hybridization signals. (**C**) Schematic representation of the results. Boxes represent the coding region of each *ALS* gene, and thick black lines represent the flanking DNA. The blue bar indicates the probe used for southern blotting. *E*, *Eco*RV restriction site.
